# Sex differences in muscle fatigue following isokinetic muscle contractions

**DOI:** 10.1038/s41598-021-87443-0

**Published:** 2021-04-14

**Authors:** Miguel Gomes, Paulo Santos, Paulo Correia, Pedro Pezarat-Correia, Goncalo V. Mendonca

**Affiliations:** 1 Neuromuscular Research Lab, Faculdade de Motricidade Humana, Universidade de Lisboa, Estrada da Costa, 1499-002 Cruz Quebrada, Dafundo Portugal; 2 CIPER, Faculdade de Motricidade Humana, Universidade de Lisboa, Estrada da Costa, 1499-002 Cruz Quebrada, Dafundo Portugal

**Keywords:** Musculoskeletal system, Physiology

## Abstract

Muscle fatigue is a limiting factor of human performance. It is unclear whether sex-based differences in fatigability exist during dynamic exercise of varying velocities of contraction. We aimed at exploring sex differences in muscle fatigue elicited by maximal isokinetic muscle contractions performed at different angular velocities. Twenty-six healthy participants (13 men: 23.2 ± 1.5; 13 women: 21.9 ± 3.0 years) were tested for concentric knee-extension at slow, moderate and fast angular isokinetic velocity (60, 180 and 300º.s^−1^, respectively), on non-consecutive days. The impact of sex on fatigue resistance and consecutive recovery for each isokinetic condition was explored by calculating the percent change in maximal voluntary isometric contraction (MVIC) and in rate of torque development (RTD), from pre- to post-isokinetic exercise (30 repetitions). The isokinetic fatigue index was also determined. No sex differences were obtained in response to isokinetic contractions completed at 60º.s^−1^. After performing muscle contractions at 300º.s^−1^, women had a significantly greater loss in MVIC than men (− 18.4 ± 5.5 vs. − 12.9 ± 3.8%; p = 0.009) and larger decreases in work output during isokinetic exercise (− 34.2 ± 8.9 vs − 27.5 ± 10.6%; p = 0.017). Recovery of initial MVIC strength was greater for women post-exercise at 180º.s^−1^ (15.6 ± 4.1% vs. 6.7 ± 9.5; p = 0.003). No differences were found between sexes in any condition for RTD from pre- to post-fatigue. These results suggest the presence of a sexually dimorphic fatigability in response to dynamic (isokinetic) contractions favouring men at higher absolute velocities of contraction.

## Introduction

Fatigue has been one of the most studied topics in human physiology and yet one of the most controversial. As a fundamental parameter describing skeletal muscle function, fatigue can be defined as a loss of force or power in response to contractile activity^1^. Several mechanisms contribute to muscle fatigue, which depend not only on the specificities of the task and muscle group, but also on the physical characteristics of the individual, including sex^2,3^. The impact of sex on fatigue is difficult to understand because the interaction between both is highly dependent on the nature of the motor task to be performed^4^.

Although some studies reported no differences in muscle fatigue between sexes, many others have shown that, regardless of being stronger than women, men are often more fatigable for sustained and intermittent isometric exercise performed at similar relative intensity^5^. Men have also been shown to recover more slowly than women following isometric exercise (sustained and intermittent), and this is likely related to heightened central fatigue in men^6,7^, thus corroborating the concept that sex differences in muscle fatigue may extend well beyond the actual exercise period. Despite the current evidence, it is still unclear whether sex-differences in fatigability are present during dynamic motor tasks performed with different muscle groups, contraction velocities and loads. In addition, it has not yet been determined which intrinsic factors may concur to a sexually dimorphic pattern during dynamic exercise. For instance, while external blood flow restriction (i.e. induced by a cuff) appears to result in sex differences in fatigability favouring women during low-intensity contractions performed with the elbow flexors^8^, this is not the case for knee-extension exercise^9^. Similar inconsistencies have been reported for isotonic contractions completed at different velocities and with different muscle groups. For instance, there are data showing that women are less fatigable than men during low-load dynamic exercise (20% of maximum voluntary isometric contraction (MVIC)) with the elbow flexors at slow, but not high-velocity contractions (i.e. ~ 60º.s^−1^ vs. maximum velocity, respectively)^10,11^. In contrast, for the knee-extensors, men show a similar reduction in maximal angular velocity as women while responding to muscle contractions performed as fast as possible at 20% MVIC. Yet, under these circumstances, men still exhibit a greater decline in MVIC torque immediately after exercise^10^.

Nevertheless, as reported for the immediate post-exercise period, women exhibit a faster rate of MVIC recovery after 120 maximal voluntary concentric contraction at 20% MVIC^7^. It was also shown that the mechanistic basis of such differences relies on sexual dimorphism at the peripheral level—with women showing contractile properties more compatible with a greater proportional area of fibers containing type I myosin heavy chain^7^.

Despite the relevance of these findings, it is important to note that past research did not control for differences in knee-extensor peak velocity between sexes (men: 420 vs. women: 290º.s^−1^), and this is an important limitation^7^. In addition, it should be emphasized that previous experimental designs focusing on dynamic muscle contractions were unable to ensure comparable conditions between sexes and this precludes drawing further conclusions. To discriminate the role of sex in muscle fatigue during dynamic exercise, it is critical to test both men and women at similar absolute angular velocities. In previous studies that explored sex differences in muscle fatigue resulting from isokinetic exercise, a single velocity of exercise was implemented (i.e. 90º.s^−1^ or 180º.s^−1^)^12–14^. While some authors found no differences in torque or work decrement between sexes after 50 continuous cycles of maximal knee-extension exercise at 90º.s^−1^^12^ and 180º.s^−1^^14^, others observed a sex difference favouring men after completing 50 repeated maximal knee-extensions at 180º.s^−1^ (when comparing the force output per unit muscle cross-sectional area from the 1st to 5th contraction)^13^. Whether a sexual dimorphic patter is present following knee-extension exercise performed at higher isokinetic angular velocity, is not known. Therefore, it remains to be unravelled whether fatigue follows a sexually dimorphic pattern in response to isokinetic muscle contractions completed at different angular velocities (i.e. slow vs. moderate vs. fast velocities).

Past research on the topic of sex differences in muscle fatigue approached this issue by quantifying the magnitude of post-exercise reduction in average/peak levels of torque, power or work output. However, one of the most critical aspects of sports performance and injury prevention is rate of torque development (RTD), which can be defined as the ability to increase torque as quickly as possible during a rapid voluntary contraction from a low or resting level^15^. Sex may influence the ability for explosive torque production because men clearly outperform women in absolute RTD^16^. Differences in absolute strength, weight-normalized tendon cross-sectional area and tendon stiffness, intrinsic contractile properties and agonist muscle activation most likely underlie this sexually dimorphic pattern^16–18^. Yet, only the first mechanism was unequivocally shown to provide a partial explanation for differences in absolute RTD between sexes^16^. When accounting for sex differences in maximal strength, men and women show similar torque-generating capacity in response explosive muscle contractions^16^. Unfortunately, to our knowledge, no previous research has compared decreases in explosive torque production between sexes after isokinetic fatiguing exercise performed at different angular velocities.

Considering all these aspects, this study aimed at determining the impact of sex on the decline, as well as on the recovery, of MVIC and RTD post-isokinetic knee-extension exercise performed at slow, moderate and fast angular velocities. We also explored sex differences in the reduction of mechanical work output (fatigue index) during isokinetic exercise performed at each angular velocity. It was hypothesized that women fatigue less than men after completing 30 maximal knee-extension isokinetic contractions at slow angular velocity. In addition, we hypothesized that both sexes exhibit similar levels of fatigue in response to 30 maximal knee-extension isokinetic contractions performed at fast angular velocity. Finally, we hypothesized that women recover at a faster rate than men after isokinetic exercise in all conditions—slow, moderate and fast angular velocities.

## Methods

### Participants

Twenty-six participants (13 men and 13 women) were included in this study (see Table 1). Physical activity levels were assessed using “The Aerobics Centre Longitudinal Study Physical Activity Questionnaire”^19^. Exclusion criteria included body mass index ≥ 25 kg.m^−2^, participation in less than 150 min of moderate to vigorous physical activity per week and also any involvement in regular resistance training (frequency ≥ 2 exercise sessions/week) for the lower limb during the past 8 weeks before volunteering for this study. Participants were tested on their dominant limb, which was determined using the Waterloo limb-dominance questionnaire^20^. All participants were healthy and free from any musculoskeletal injury that would limit exercise performance. The risks of participation were explained and informed consent was obtained from all participants. This study complied with the principles set forth in the Declaration of Helsinki and was approved by the Faculty’s Ethics Committee (CEFMH nº: 15/2019).

**Table 1:** Characteristics of the participants.

	Women (n = 13)	Men (n = 13)	p value
Age (years)	21.9 ± 3.0	23.2 ± 1.5	0.088
Height (cm)	162.8 ± 6.6	175 ± 6.8	< 0.001*
Body mass (kg)	58.2 ± 6.0	73.7 ± 10.9	< 0.001*
BMI (kg/m^2^)	22 ± 2.0	24 ± 3.1	0.061
PA (MET-h/wk)	41.3 ± 4.2	40.6 ± 5.0	0.701

Values are mean ± SD.

*BMI* body mass index, *PA* physical activity, *MET* metabolic equivalent.

*Sex difference at p < 0.05.

### Procedures

Each participant visited the laboratory on 4 different non-consecutive days to complete 1 familiarization session and 3 testing sessions (one at each angular velocity on a randomized fashion). All sessions were conducted between 12:00 and 17:00 h. Participants were tested for unilateral knee-extension exercise. They all completed the following tasks on each testing session: (1) knee-extension MVICs (2) isokinetic knee-extension fatigue protocol and (3) post-fatigue MVICs. Participants were also asked to avoid the consumption of alcohol, xanthine derivatives and engagement in any form of strenuous lower-limb exercise 24 h before testing (24, 12 and 48 h, respectively).

### Measurements

Participants remained seated on a Biodex System 3 Pro isokinetic dynamometer (Biodex Medical Systems, Shirley, NY) with a hip angle of 85º (supine position = 0º). For each knee-extension MVIC, the knee joint was fixated at 70º of knee extension^21^. For the isokinetic fatigue protocol, knee-extension range of motion was set at 90º (0º = maximum knee-extension). All torque readings were corrected for the effect of gravity on the lower limb in accordance with the manufacturer recommendations. Velcro straps were placed across the trunk, hip and thigh to prevent extraneous movement. The axis of rotation of the dynamometer was aligned with the lateral epicondyle of the knee. The lower leg was also strapped to the knee extension/flexion attachment, which was placed at a standardized distance of 3 cm from the medial malleolus. Torque signal was obtained at 1000 Hz (MP150, BIOPAC Systems Inc., Goleta, CA). Data were collected and processed using the software AcqKnowledge 4.3.1 (BIOPAC Systems Inc., Goleta, CA). A 12 Hz low-pass filter (zero-phase shift 4th order Butterworth filter) was applied to torque signals, using a custom-built routine for analysis (MATLAB version R2018a).

### Protocol

Two days before testing, each participant underwent a familiarization session during which additional information was provided, along with completion of the questionnaires. Afterwards, the participants were submitted to a familiarization protocol including both isometric and isokinetic contractions performed at three different angular velocities (60, 180 and 300º.s^−1^). This was done to minimize the learning effect associated with this specific motor task^22^.

Each testing session began with a dynamic warm up, consisting of 5 min of submaximal cycle-ergometry set at 25 W. Then, participants performed two sets of 5–6 submaximal isokinetic repetitions (1 set at 120º.s^−1^ and 1 set at speed test: 60, 180 or 300º.s^−1^) with 30 s of pause between sets. This was followed by 4–5 submaximal isometric repetitions (with the knee at 70º of extension) at ~ 60–70% of participants’ perceived maximum effort. The last repetition corresponded to a 5-s MVIC to promote post-activation potentiation^23^. A rest period of 4 min was allowed between the completion of warm up and testing procedures. Four maximal isometric 4-s voluntary knee extensions were then performed, with 1 min rest between trials. The participants were instructed to exert their maximum force “as fast and hard as possible”, to obtain both maximal torque and RTD^15^. To ensure an accurate assessment of these variables, visual instantaneous feedback of the torque-time curve was provided to all participants during each trial. MVIC was defined as the single highest peak torque (PT) data point obtained during these isometric contractions. The highest PT value from the four MVIC’s was used as a measure of maximum isometric strength pre-fatigue (baseline). Another five maximal voluntary knee extensions were performed post-fatigue, with 1 min of interval between trials. The 1st (performed immediately post-exercise cessation) and 5th repetition (post-fatigue.1 and post-fatigue.5, respectively) were then used to explore the magnitude of post-exercise recovery.

RTD was computed using different approaches. Sequential RTD was calculated using the torque-time curve slope (i.e. Δtorque/Δtime) and analysed in incremental epochs of 50 ms (0–50; 50–100; 100–150 ms). Peak RTD (pRTD), which corresponds to the highest torque-time curve slope, was calculated using 20-ms time windows^15^. Explosive torque (ETorque) was defined as the %MVIC attained at specific time points (50, 100, 150 and 200 ms). It represents a relative measure of explosive torque production and it translates the ability to recruit the individual torque reserve. The onset of torque development (start of contraction) was defined as the time point at which the torque curve exceeded the average baseline values by 3 N.m^15,24^. Contractions associated with pre-tension or counter-movement were discarded, and another trial was performed. Torque, pRTD and sequential RTD were measured in absolute and normalized terms (relative to MVIC) i.e. relative pRTD was calculated as follows: $\frac{\mathrm{pRTD}({\mathrm{N}.\mathrm{m}.\mathrm{s}}^{-1})\times 100}{\mathrm{MVIC}(\mathrm{N}.\mathrm{m})}$

For the isokinetic muscle contractions, testing involved a fatigue protocol consisting of 30 maximal repetitions performed in the concentric/passive mode at randomly pre-selected angular velocities—slow, moderate and fast (60, 180 and 300º.s^−1^, respectively)^25^. Passive mode velocity was set at 90º.s^−1^ for all conditions. Participants were instructed to exert maximal torque as fast and hard as possible during the concentric phase, corresponding to knee extension. Knee flexion was performed passively. The impact of sex on fatigue resistance at each isokinetic angular velocity was explored by calculating the percent change in MVIC and pRTD across time points (fatigue: MVIC_loss_ and pRTD_loss_—from baseline to post-fatigue.1; recovery: MVIC_rec_ and pRTD_rec_–from post-fatigue.1 to post-fatigue.5). Analysis on the impact of fatigue at the level of sequential RTD and ETorque were exclusively performed in transition from baseline to post-fatigue.1. Finally, we computed the modified isokinetic fatigue index for PT and work output to explore sex differences in the decline of muscle performance during isokinetic torque production:

1 $$Fatigue\, index \left(\% decrease\right)=\frac{\left(\stackrel{-}{x} 5\, highest\, consecutive\, repetitions-\stackrel{-}{x} last\, 5\, repetitions\right)}{\stackrel{-}{x} 5\, highest\, repetitions}\times 100$$

in which, $\stackrel{-}{x}$ represents the mean value of PT or work output. This equation has been shown to be more accurate than the traditional isokinetic fatigue index (which accounts for the first five repetitions instead of the highest consecutive five repetitions)^26^. Work output was calculated as the area under the torque–angle curve during the isokinetic window of each velocity (angular acceleration = 0).

### Statistical analysis

**Figure 1:**
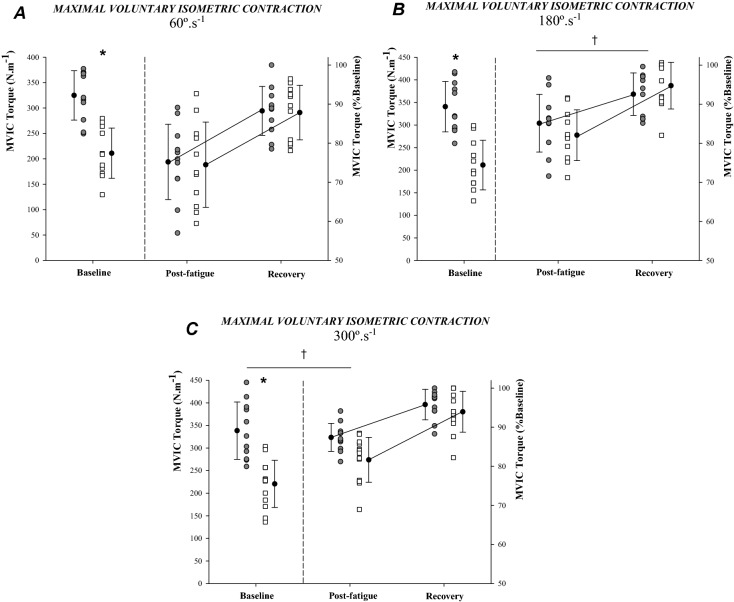
Maximal voluntary isometric contraction (MVIC) at pre-, post-exercise and during recovery from isokinetic knee-extensions performed in each condition (**A**: 60º.s^−1^; **B**: 180º.s^−1^; **C**: 300º.s^−1^). Men are represented by the grey (filled) circles and women by the white squares. Baseline, measurements were taken before exercise; post-fatigue.1 measurements were taken immediately after exercise and post-fatigue.5 measurements were taken 5 min after exercise cessation. *Sex difference at specific time-point (p < 0.05); † sex difference in the delta between time-points (fatigue or recovery) (p < 0.05).

**Figure 2:**
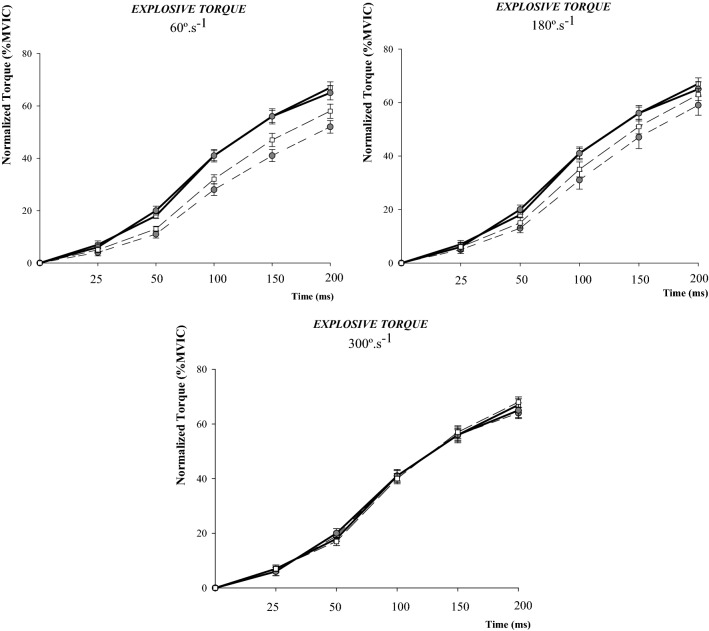
Comparison of explosive torque between men and women from pre- to post-exercise in each condition (**A**: 60º.s^−1^; **B**: 180º.s^−1^; **C**: 300º.s^−1^). Men are represented by the grey (filled) circles and women by the white squares. Pre- exercise data are represented by the continuous line and post-exercise data are represented by the dash line. *MVIC* maximal voluntary isometric contraction.

**Figure 3:**
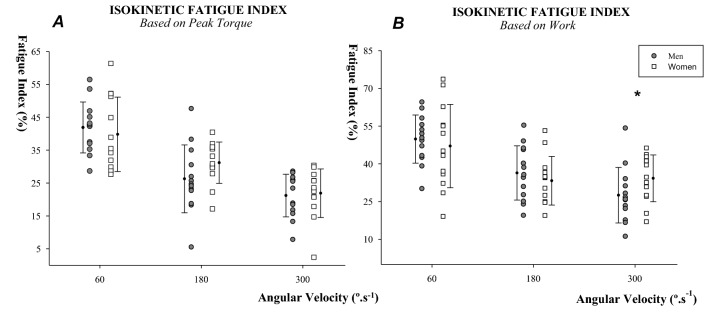
Isokinetic fatigue index based on peak torque (**A**) and work output (**B**). Men are represented by the grey (filled) circles and women by the white squares. *Sex difference at (p < 0.0).

Descriptive and outcome statistics are presented as mean ± standard deviation (SD) in the text and Figs. 1 and 3 and as mean ± standard error of the mean (SEM) in Fig. 2. Leg dominance was compared between sexes with the Mann–Whitney U, non-parametric test. Cohen’s *d* effect-size analysis was used to determine the proportion of total variance that is attributable to sex differences for torque-related variables. Based on previous research, if the decrement in MVIC from pre- to post-dynamic knee-extension fatiguing tasks in men corresponds to 35.0 ± 13.4% and 23.1 ± 8.4% in women^7,26^, a sample size of 24 participants (12 men and 12 women) was estimated to achieve more than 80% power of correctly rejecting the null hypothesis. Therefore, 26 participants were recruited for this study. Independent samples *t* tests were used to explore sex differences in anthropometric characteristics, physical activity levels and in baseline measures of torque-related variables. Separate repeated-measures two-way ANOVAs, with sex as a between-subject factor (males vs females), were computed to compare changes in MVIC and pRTD over time (fatigue and recovery) during each testing session (60, 180 and 300º.s^-1^). To assess the time effect of fatigue elicited by the dynamic muscle contractions, we compared data obtained at baseline with those obtained immediately after the cessation of exercise (baseline vs. post-fatigue.1). Additionally, we also explored between-sex differences in the magnitude of post-exercise recovery. This was done by comparing the time point immediately subsequent to the fatiguing task with that seen after 5 min of recovery (post-fatigue.1 vs. post-fatigue.5). Post hoc analyses were performed using independent samples *t* tests, with sex as the grouping variable. For variables calculated only at baseline and post-fatigue.1 (i.e. sequential RTD and ETorque), independent samples *t* tests (with sex as the grouping variable) were used to determine sex differences between time-points. To analyse changes resulting from the isokinetic fatiguing task, repeated-measures two-way ANOVAs were separately computed for PT and Work fatigue indexes (with sex [2] and velocity [3] as between- and within-subject’s factors, respectively). Post-hoc analyses were performed by means of independent samples *t* tests, with sex as the grouping variable. All data were tested for normality with the Kolmogorov–Smirnov test. For each ANOVA, data were tested for sphericity with Mauchly’s test. For independent-samples t tests, the Levene’s test for equality of variances was performed. Data were analysed using IBM SPSS Statistics (IBM Corp. Released 2011. IBM SPSS Statistics for Windows, Version 25.0. Armonk, NY: IBM Corp.) and significance was set p < 0.05.

### Ethics approval and consent to participate

Ethical approval for this study was obtained from Conselho de Ética da Faculdade de Motricidade Humana (CEFMH nº: 15/2019).

## Results

### Demographics and baseline measures

As shown in Table 1, men and women were of similar age and attained similar levels of weekly physical activity (p > 0.05). They did not differ for body mass index, however men were heavier (sex main effect, F = 20.0, p < 0.001) and taller than women (sex main effect, F = 21.1, p < 0.001). There were no sex-differences in leg dominance (p > 0.05). Tables 2, 3 and 4 depict the differences between sexes in torque-related variables at each angular velocity. Overall, knee-extension absolute MVIC torque was 34% higher in men compared to women (men: 330.5 ± 43.6 vs. women: 216.6 ± 49 N.m; sex main effect, F = 29.1, p < 0.001, *d* = 2.2). Men also exhibited 38% higher levels of absolute pRTD (1750.3 ± 330.6 vs. 1077.2 ± 267.3 N.m.s^−1^; sex main effect, F = 30.9, p < 0.001, *d* = 2.2). Finally, they also showed enhanced absolute values of sequential RTD (50 ms epochs) (40% difference RTD_0–50_, *d* = 1.7; 35% difference RTD_50–100_ *d* = 1.7; 30% difference RTD_100–150_*, d* = 1.6; sex main effect, F = 30.6, p < 0.001). However, after normalizing pRTD and sequential RTD to MVIC, sexual dimorphism in all these variables was dissipated (p > 0.05, for all comparisons). No sex differences were found for ETorque across time points in either condition (p > 0.05).

**Table 2:** Relative change in mechanical variables from pre- to post-isokinetic contractions and during recovery from exercise performed at different angular velocities.

Variables	60º.s^−1^		180º.s^−1^		300º.s^−1^	
	Men	Women	Men	Women	men	Women
MVICloss (%)	− 24.8 ± 9.19 *(*− *31.2 , *− *18.3)*	− 27.2 ± 10.4 *(*− *33.8 , *− *27.2)*	− 15.2 ± 6.85 *(*− *20.1 , *− *9.8)*	− 18.8 ± 10.4 *(*− *22.6 , *− *14.9)*	− 12.9 ± 3.81 *(*− *15.9 , *− *10.5)*	− 18.4 ± 5.52* *(*− *21.8 , *− *14.9)*
MVICrec (%)	16.1 ± 7.3 *(10.9 , 21.2)*	17.6 ± 9.3 *(11.7 , 23.4)*	6.7 ± 9.5 *(3.6 , 14.5)*	15.6 ± 4.12* *(12.9 , 18.2)*	8.03 ± 6.6 *(4.3 , 14.6)*	13.04 ± 6.4 *(9.1 , 17.2)*
Absolute pRTDloss (%)	− 41.8 ± 21.2 *(*− *56.7 , *− *26.8)*	− 39.3 ± 19.8 *(*− *51.8 , *− *26.8)*	− 22.3 ± 25 *(*− *38.6 , *− *17.1)*	− 26.5 ± 11.9 *(*− *34.1 , *− *18.9)*	− 15.8 ± 15.8 *(*− *28.1 , *− *11.4)*	− 12.6 ± 14.5 *(*− *21.7 , *− *3.5)*
Absolute pRTDrec (%)	22.6 ± 28.8 *(2.3 , 42.8)*	21.3 ± 29.8 *(2.5, 40.1)*	12.5 ± 22.5 *(2.5 , 40.1)*	16.3 ± 14.6 *(7.1 , 25.5)*	− 2.3 ± 30.6 *(*− *20.7 , 18.1)*	4.33 ± 9.5 *(*− *9.6 , 18.3)*
Relative pRTDloss (%)	− 235 ± 125.6 *(*− *323 , *− *146)*	− 207 ± 105.6 *(*− *269 , *− *141)*	− 135 ± 113.3 *(*− *205 , *− *63)*	− 135 ± 67.7 *(*− *177 , *− *92)*	− 82 ± 85.9 *(*− *135 , *− *27)*	− 86 ± 105.7 *(*− *152 , *− *19)*
Relative pRTDrec (%)	105 ± 103.2 *(32 , 177)*	88.8 ± 105.7 *(18 , 158)*	74 ± 105.4 *(2 , 150)*	74 ± 67.7 *(23 , 112)*	16.4 ± 125.9 *(*− *49 , 99)*	36 ± 71.7 *(16 , 86)*

Values are mean ± SD and 95% *confidence intervals.*

*MVIC*_*loss*_ relative change in maximal voluntary isometric contraction from baseline to immediately after fatigue, *pRTD*_*loss*_ relative change in peak rate of toque development from baseline to immediately after fatigue, *MVIC*_*rec*_ relative change in maximal voluntary isometric contraction from immediately after fatigue to 5-min post-exercise cessation, *pRTD*_*rec*_ relative change in peak rate of torque development from immediately after fatigue to 5-min post-exercise cessation.

*Sex difference at p < 0.05.

**Table 3:** Comparison of absolute rate of torque development from pre- to post- fatigue in both sexes.

Variables	*60º.s*− ^*1*^							
	Pre-exercise		Post-exercise		p value	∆ (%)		
Absolute RTD (N.m/s− ^1^)	Men	Women	Men	Women		Men	Women	P value
0–50 ms	1310 ± 342	787 ± 197	548 ± 168	384 ± 100	< 0.001*	− 53.9 ± 19.9	− 48.8 ± 15.4	0.500
50–100 ms	1378 ± 318	951 ± 206	826 ± 247	565 ± 109	< 0.001*	− 38.1 ± 17.4	− 37.7 ± 18.9	0.956
100–150 ms	932 ± 238	619 ± 112	620 ± 0.256	477 ± 124	< 0.001*	− 30.5 ± 27.7	− 22.7 ± 16.7	0.447

Variables	*180º.s*− ^*1*^							
	pre-exercise		post-exercise		p value	∆ (%)		
Absolute RTD (N.m/s^−1^)	Men	Women	Men	Women		Men	Women	P value
0–50 ms	1191 ± 354	722 ± 274	704 ± 341	473 ± 274	< 0.00*	− 48.3 ± 2.7	− 22.6 ± 43.7	0.095
50–100 ms	1431 ± 363	924 ± 294	1033 ± 471	687 ± 182	< 0.001*	− 28.9 ± 29.9	− 21.5 ± 22.1	0.503
100–150 ms	949 ± 141	679 ± 145	888 ± 268	569 ± 122	0.055	− 3.76 ± 31.6	− 14.7 ± 16.4	0.299

Variables	*300º.s*− ^*1*^							
	Pre-exercise		Post-exercise		p value	∆ (%)		
Absolute RTD (N.m/s^−1^)	Men	Women	Men	Women		Men	Women	P value
0–50 ms	1292 ± 347	757 ± 0.26	1109 ± 377	567 ± 115	< 0.001*	− 13.6 ± 19.1	− 17.8 ± 26.3	0.660
50–100 ms	1473 ± 304	925 ± 226	1342 ± 291	838 ± 164	0.006*	− 7.9 ± 13.7	− 7.39 ± 14.0	0.918
100–150 ms	924 ± 187	658 ± 172	893 ± 191	615 ± 162	0.188	− 1.7 ± 18.1	− 5.3 ± 17.2	0.626

Values are mean ± SD.

*RTD* Rate of torque development.

*Difference from pre- to post-exercise at p < 0.05.

**Table 4:** Comparison of normalized values of sequential rate of torque development from pre- to post-fatigue in both sexes.

Variables	*60º.s*− ^*1*^							
	Pre-exercise		Post-exercise		p value	∆ (%)		
Relative RTD (%MVIC)	Men	Women	Men	Women		Men	Women	p value
0–50 ms	412 ± 116	376 ± 101	237 ± 98	257 ± 74	< 0.001*	− 160.3 ± 133	− 119.7 ± 87.3	0.394
50–100 ms	422 ± 65	445 ± 54	337 ± 72	373 ± 72	< 0.001*	− 77.5 ± 103	− 72.2 ± 78.2	0.889
100–150 ms	286 ± 67	294 ± 53	249 ± 74	313 ± 75	0.644	-34.1 ± 79.6	18.8 ± 44.4	0.069

Variables	*180º.s*− ^*1*^							
	Pre-exercise		Post-exercise		p value	∆ (%)		
Relative RTD (%MVIC)	Men	Women	Men	Women		Men	Women	p value
0–50 ms	370 ± 123	342 ± 105	255 ± 120	290 ± 140	0.002*	− 114 ± 106	− 51.6 ± 129	0.204
50–100 ms	430 ± 83	434 ± 79	369 ± 142	403 ± 71	0.096	− 60 ± 173.2	− 30.5 ± 73.8	0.584
100–150 ms	290 ± 50	326 ± 60	318 ± 83	335 ± 49	0.247	29 ± 87.9	8.9 ± 69.7	0.542

Variables	*300º.s*− ^*1*^							
	Pre-exercise		Post-exercise		p value	∆ (%)		
Relative RTD (%MVIC)	Men	Women	Men	Women		Men	Women	p value
0–50 ms	396 ± 115	352 ± 114	380 ± 105	334 ± 110	0.297	− 15.4 ± 76.1	− 17.7 ± 79.2	0.944
50–100 ms	443 ± 59	424 ± 65	463 ± 62	473 ± 50	0.008*	19.9 ± 62.1	49.2 ± 53.1	0.227
100–150 ms	282 ± 68	301 ± 48	311 ± 69	345 ± 59	0.001*	28.7 ± 46.4	44.1 ± 49.1	0.435

Values are mean ± SD.

*RTD* Rate of torque development.

*Difference from pre- to post-exercise at p < 0.05.

Baseline data (MVIC, pRTD, sequential RTD and ETorque), obtained in each condition, were similar between angular velocities (60, 180 and 300º.s^−1^). Importantly, this occurred similarly for both men and women.

### Isometric torque production (fatigue and recovery)

#### MVIC and pRTD

Data for MVIC and pRTD are presented in Table 2 and Fig. 1. For all conditions, in both sexes, MVIC and pRTD were reduced from baseline to immediately post-isokinetic exercise (MVIC_loss_; time main effect, F = 181.8, p < 0.001; pRTD_loss_; time main effect, F = 48.8, p < 0.001). Then, MVIC increased throughout the 5 min of recovery (MVIC_rec_; time main effect, F = 103.0, p < 0.001; pRTD_rec_; time main effect, F = 10.1, p = 0.004) (Table 2). Although MVIC_loss_ and MVIC_rec_ were similar between men and women at 60º.s^−1^, significant interactions were obtained at 180 and 300º.s^−1^ (sex × time interaction, F = 6.7, p = 0.016 and F = 6.1, p = 0.021, respectively). For MVIC_loss_ at 180º.s^−1^, while both sexes had identical decrements over time (p = 0.187), women had a greater MVIC_rec_ than men (p = 0.007, *d* = 1.2). Additionally, at 300º.s^−1^ women had a greater MVIC_loss_ than men (p = 0.009, *d* = 1.1), while MVIC_rec_ was statistically similar for both sexes (p = 0.186) (Fig. 1). The pRTD_loss_ and pRTD_rec_ response was similar between sexes in all conditions both for absolute and normalized values (p > 0.05).

#### Sequential RTD and ETorque

Absolute and normalized values of sequential RTD and ETorque are shown in Tables 3 and 4, as well as in Fig. 2, respectively (these variables were only calculated for fatigue). Isokinetic knee-extension exercise performed at all angular velocities (60, 180 and 300º.s^−1^) was effective in reducing sequential RTD (both absolute and normalized values) and ETorque in both sexes. However, this was not extensive to all time intervals (see Tables 3 and 4). Comparisons between sexes revealed that the magnitude of change in sequential RTD_loss_ (absolute and normalized) and ETorque_loss_ was statistically similar between men and women in all conditions (p > 0.05), despite the moderate effect size (*d* = 0.2–0.8) indicating a smaller decrease in most time intervals favouring women.

#### Fatigue during isokinetic exercise

During isokinetic exercise, dynamic PT and work output decreased from the start to the end of the protocol in all conditions (Fig. 3). No significant interactions between sex and velocity were found for PT fatigue index during isokinetic exercise (p = 0.172). However, for the Work-based fatigue index, a significant interaction was obtained (sex × velocity interaction, F = 6.0, p = 0.005). Post-hoc analyses revealed that, although no differences between sexes were found at 60 or 180ºs^−1^ (p > 0.05), women fatigued 7% more than men while responding to the exercise performed at 300ºs^−1^ (women: 34.2 ± 8.9 vs. men: 27.5 ± 10.6%; p = 0.017, d = 0.7) (Fig. 3).

## Discussion

The aim of this study was to explore whether sex differences in muscle fatigue are sustained during and after maximal isokinetic knee-extension exercise performed at different angular velocities. Our results indicate that different velocities of contraction have a distinct impact on muscle performance, and that sex interacts with the relationship between contraction velocity and muscle fatigue. We unravelled that men exhibit smaller work output decrement and better isometric muscle performance post-fatigue induced by isokinetic knee extensions performed at faster angular velocities (300º.s^−1^). Additionally, we found that women only recover faster than men after isokinetic exercise completed at moderate velocities of contraction (180º.s^−1^). Taking into consideration that both sexes exercised at similar absolute angular velocities, these findings are in partial agreement to that hypothesized.

We chose an isokinetic paradigm to investigate muscle fatigue because, under these conditions, muscle contractions are performed at maximal intensity throughout the entire isokinetic range of motion, regardless of the selected velocity^12^. Despite being a non-natural condition for real-life motor performance, it provides insight into the individual single-limb exercise capacity at maximal intensity, but limited velocity. Also, we selected an exercise approach involving similar absolute velocities between sexes because isokinetic testing (i.e. agonist/antagonist ratios and fatigue indexes), training and rehabilitation are typically performed using standardized absolute, rather than relative velocities^27^. As confirmed by our data, under these circumstances, muscle fatigue manifests itself differently from that seen in prior research with isotonic contractions in both sexes^9,12^.

### Isokinetic and isometric fatigue

#### MVIC, dynamic PT and work output

First, since MVIC and RTD decreased from pre- to post-exercise in all conditions, it can be confirmed that isokinetic contractions were effective in eliciting muscle fatigue (loss of torque/power)^1^. From an absolute-velocity analytical standpoint, our data provide evidence that there is no sexual dimorphism in torque decrement or muscle performance in response to isokinetic fatiguing exercise performed at slow velocity (60º.s^−1^). In line with our findings, previous reports have shown no sex differences in PT decrease or relative work output when performing 150 and 50, respectively, isokinetic knee-extensions at 90º.s^−1^^28^. Contrary to that seen in the present study using isokinetic contractions, it has been shown that during isotonic slow-velocity contractions (~ 60º.s^−1^) women are more fatigue-resistant than men^9^. Additionally, corroborating these data, women have been shown to attain a longer time to failure in response to low-intensity dynamic elbow-flexion exercise (20% MVIC)^11^. It is generally believed that this is secondary to sex-related differences in contractile mechanisms regulating the changes in peak rates of muscle relaxation^17^. However, there is undisputed evidence that maximal knee-extension angular velocity is higher in men than women^7^. This is relevant because the available research indicates that the recruitment of type II muscle fibers varies as a function of relative angular velocity^29,30^. Hence, at 60º.s^−1^, the typical female-associated reliance on type I fibers for muscular work^31,32^ was likely offset because women contracted at a higher relative velocity than men. Altogether, these data strongly suggest that the naturally occurring sex differences in maximum angular velocity likely explain the absence of sexual dimorphism in muscle fatigue post-isokinetic knee-extension exercise performed at 60º.s^−1^.

As above-mentioned, not many previous experimental designs have controlled for contraction velocity, especially during isotonic contractions (i.e. slow velocity was set for 60º.s^−1^ and fast velocity for “as fast as possible”). Few studies have also explored sex differences at moderate velocities of contraction, in this case 180º.s^−1^. Our data support those of past studies showing that no sex differences exist in muscle fatigue during isokinetic exercise at slow and moderate velocities^12,14,28^ and contrast with the findings of others (analytical perspective examining sex differences in fatigue post-exercise performed at similar absolute velocity between sexes)^26,33^. Wretling & Henriksson-Lársen (1998) found no differences between men and women in mechanical output following 150 knee-extension isokinetic contractions at 90º.s^−1^. In contrast, Pincivero et al. (2000) found a sexually dimorphic pattern in peak work output favouring women after 30 maximal isokinetic knee-extensions performed at 180ºs.^−1^. Unfortunately, the authors did not normalize their data to MVIC and this limits further interpretations. In another study, the same authors reported a higher rate of quadriceps femoris muscle fatigue in men than in women. Fatigue was calculated in response to 30 isokinetic knee-extensions at 180º.s^-1^ using the modified fatigue index^26^. Even though our methods were relatively similar to those of past reports (number of repetitions, angular velocity, isokinetic variable and fatigue quantification), we found no sex differences in fatigue index for PT or work output. We therefore provide preliminary evidence that the magnitude of MVIC reduction is not different between sexes after isokinetic exercise at 180ºs^−1^ (moderate velocity). However, from the perspective of relative angular velocities (as previously described in this section), it should be noted that women exhibited similar fatigue resistance as men despite having exercised at a higher percentage of their maximal velocity. This greater capacity of women to delay the onset of fatigue while performing muscle contractions at higher relative velocities is potentially due to heightened vasodilation in response to a varied spectrum of exercise intensities^34,35^, which causes a state of enhanced blood supply to the working muscles.

Contrary to what has previously been reported in the literature, our data suggest that men are less fatigable than women during and after isokinetic exercise completed at an angular velocity of 300º.s^−1^. Some studies reported that women exhibit less fatigue than men at high velocities of contraction for the knee extensors^7,10^, but not for the elbow flexors^10^. Our data show that men are more resistant than women during isokinetic exercise at 300º.s^−1^ (7% less decrease in work output). They also show that men experience an attenuated reduction in MVIC following this specific motor task (5.5% less fatigue). However, when interpreting our data, it is relevant to consider that both sexes exercised at the same absolute isokinetic velocity. There is a great level of inconsistency between studies at this particular level and this is likely secondary to the methodological differences of past reports. For instance, in the study conducted by Senefeld et al. (2018), the participants were asked to perform the task “as fast as possible”, resulting in greater velocities of isotonic maximal concentric contractions for men than women (men: 420 vs. women: 290º.s^−1^). All women included in the present study were able to achieve the pre-defined angular displacement (i.e. isokinetic window) during isokinetic exercise performed at 300º.s^−1^. Therefore, they all performed below their knee-extension maximal angular velocity. Yet, based on the concept that knee-extension maximal angular velocity is different between sexes^7^, it can be assumed that women exercised closer to their maximal angular velocity during muscle contractions performed at 300º.s^−1^. This indicates that, when exercising at an absolute intensity closer to their maximal angular velocity (i.e. at 300º.s^−1^), women experience greater fatigue. To our knowledge, this is the first study to explore sex differences in muscle fatigue using high-velocity isokinetic exercise. Because the concentric phase at 300ºs^−1^ only lasts 0.3 s, the greater ability of men to produce torque under a time constraint (and consequently attain higher velocities of contraction than women) might reside in differences at the level of the rate-limiting mechanisms of maximal velocity of fiber contraction (speed of cross-bridge cycling and Ca^2+^ kinetics in the fiber)^1^. It has been shown that the potentiation of twitch torque in response to maximal muscle contraction is greater in men compared to women, irrespectively of age^36^. This corroborates the notion that men exhibit a greater increase in actin-myosin Ca^2+^ sensitivity in response to high intensity muscle contractions^36^. Moreover, in women, contractile properties are typically characterized by longer half-relaxation time and lower evoked-twitch torque than men^36,37^. This slowing is consistent with women exhibiting a higher level of type I fibers^38^ involved in torque production throughout the entire range of motion (work). The sexual dimorphism in phenotypic muscle fiber-type expression might explain why the relationship between absolute and relative angular velocities differs between men and women. Understanding why men are more capable of resisting fatigue during fast-velocity isokinetic contractions becomes intuitive based on the premise that, while exercising at a given absolute angular velocity, men perform at at a lower relative velocity than women. As it is well known, exercising at higher relative velocities implicates the additional recruitment of high-threshold motor units, in which type II muscle fibers predominate (with higher rates of force production and relaxation)^3,39^. It is possible to assume that the phenotypic traits associated with muscle fiber type expression in women might exert a negative impact in their ability to delay fatigue in response to high-velocity contractions. Since our data indicate a statistically significant sex difference in MVIC decrement of 5.5%, this may well be the case.

#### pRTD, sequential RTD and ETorque

This is the first study that explored sex differences in RTD following the onset of fatigue elicited by isokinetic exercise performed at different velocities. Our data show that each velocity exerts a different impact in the ability to produce torque rapidly because greater decrements were found for both sexes at 60º.s^−1^ compared to 180 s^−1^ and 300º.s^−1^. (Tables 2, 3 and 4). We also explored whether RTD is differently affected between sexes in response to each condition (velocity). We found that the fatigue-induced reduction of pRTD, sequential RTD and ETorque does not follow a sexually dimorphic pattern post-isokinetic exercise performed at 60, 180 or 300º.s^−1^. Importantly, this was sustained when data were analysed both in an absolute and normalized terms.

Explosive torque was here defined as the ability to increase torque as quickly as possible during a fast voluntary contraction from a low or resting level^15^. Our findings are in accordance with those of Hannah et al. (2012), thus corroborating the concept that both sexes have similar ability to express the available torque-generating capacity, despite men outperform women in absolute values.

Neural determinants, intrinsic contractility and contractile capacity assume different preponderance during early and late stages of explosive torque production^15,18^. Previous studies examining explosive knee-extensor torque production found that, in absolute terms, agonist electromyographic (EMG) activation is a major determinant from the early to mid-phase of torque production (up to ~ 50 ms)^15,18^. Conversely, contractile properties are more closely related with torque production from 50 to 100 ms and MVIC largely determine torque-generating capacity beyond 75 ms^18^. It is interesting to note that, despite both sexes exhibit a dissimilar reduction in MVIC and mechanical work output after completing high-velocity isokinetic exercise, this does not seem to impair fast torque production to a different extent between men and women in either condition (post-30 repetitions at 60, 180 or 300º.s^−1^). Previous studies have related the decline in explosive torque production with a reduction of EMG activity^18,40^. Since agonist EMG is an important determinant of explosive torque^18^, the lack of sex differences in neural activation following the completion of the fatiguing dynamic protocols may possibly explain why men and women have similar losses in pRTD, sequential RTD and ETorque, regardless of the contraction velocity. Yet, once again, it should be reinforced that each absolute velocity required women to exercise at a higher percentage of their maximal angular velocity compared to men. Alternative conditions, such as matching both sexes for knee-extension relative angular velocity might produce different results (i.e. attenuated decrement in rapid torque production in women). This is partly supported by the moderate to large effect sizes obtained in our data when comparing differences in sequential RTD (absolute and normalized) and ETorque decrements between sexes, indicating a general smaller decrease across all velocities in women, despite the lack of statistical significance.

#### Post-exercise recovery

The present findings are in line with that reported in the existent literature supporting that women recover to a greater extent than men^7,41^. However, this was only seen at moderate velocities. Women have consistently been shown to recover at a faster rate than men following isometric (sustained and intermittent)^7,32^ and dynamic contractions^7,11^. Yet, the mechanisms of recovery differ depending on the type of contraction elicited during exercise. While central mechanisms of fatigability are more strongly associated with sexual dimorphism in recovery from isometric contractions, contractile mechanisms and differences in metabolic substrate largely explain why men recover slower than women following dynamic contractions^7^. These differences in metabolic substrate utilization resulting from muscle fatigue have been previously described by Kent-Braun et al. (2012). The authors found that during an exercise bout of increasing intensity, women have smaller increases in inorganic phosphate (Pi) and dihydrogen phosphate (H_2_PO_4_^−^) and less decrease in intracellular pH than men^7,36^. This indicates that women rely more on oxidative pathways for the supply of adenosine triphosphate for muscle contractions^31^. The greater accumulation of the aforementioned metabolic by-products (Pi, H_2_PO_4_^−^, hydrogen ions (H^+^)) in men most likely impairs their ability to recover faster because of a greater demand for metabolite clearance. However, our results do not reveal a sex difference in recovery at 60º.s^−1^ and 300º.s^−1^ and, as mentioned before, this is may be related to the differences in relative velocity between men and women for each condition.

## Perspectives and significance

This is the first study to compare muscle fatigue and recovery between men and women following isokinetic exercise performed at different angular velocities. Our results show that, when compared at the same absolute angular velocity, young men and women fatigue to a similar extent during slow and moderate isokinetic velocities. In contrast, at high contraction velocities, there is a sex difference in muscle fatigue favouring men. Rate of torque development was similarly affected in both sexes across all conditions. In addition, women recovered at a faster rate than men when exercising at moderate isokinetic velocities, confirming previous results. These findings corroborate the notion that sexual dimorphism in muscle fatigue is dependent on the specificities of the motor task and on the velocity of contraction. We also provide an important insight into sex differences in neuromuscular function and fatiguing exercise and this may be of translational value for training and rehabilitation. In specific, according to our data, at higher absolute isokinetic velocities men have to perform more repetitions than women to achieve a certain level of fatigue. Conversely, women need less time to recover than men after moderate and fast isokinetic exercise. This urges for the need of a sex-based individualization of isokinetic exercise prescription and recovery.

## Limitations

Our study has at least five important limitations. First, we did not control for the impact of the menstrual cycle on the outcome variables. There is little evidence that the menstrual cycle and related monthly fluctuations in sex hormones negatively impact performance in fatigability of young women^42^. Thus, we believe this limitation is unlikely to have affected our results. Second, the participants recruited for this study were undergraduate Sports Science students. Some of them reported to be assiduous (although amateur) practitioners of activities that require fast and explosive knee-extension movements (e.g. soccer). However, the number of male and female practitioners in this experimental design was very similar and therefore we do not believe that this limitation affected the key aspects of our study. Third, we were unable to record surface EMG from the quadriceps femoris of the participants. The ability for maximal and explosive muscular contractions is highly dependent on the neural drive to the agonist muscles^18^, and the presence or absence of sex differences in agonist EMG activation following isokinetic fatigue at different angular velocities could provide an insight into the potential mechanisms that may explain our results. Therefore, this is an important limitation to our study. Fourth, we did not measure maximal angular velocity during unloaded knee-extension exercise. This would have been important to account for differences in peak angular velocities between sexes. However, there is strong evidence that, due to differences in phenotypic muscle fiber-type expression^39^ and rate-limiting mechanisms of maximal velocity fiber contraction^1^, men outperform women in contraction velocity and consequently angular velocity^7^. Therefore, we can assume that for each angular velocity used in this study, men exercised at a lower percentage of their maximal angular velocity compared to women.

Past research has demonstrated increased central fatigue of men compared to women^6,7^. It is also known that neural determinants play a significant role in the early stage of torque production^15^. In our study, it is possible that greater central fatigue in men has negatively impacted them to a greater extent than women during the first 50 ms of contraction, as indicated by the moderate to large effect size for RTD measurements at 60º and 180º.s^−1^. Considering that RTD measurements are less reliable than MVIC^15^, the absence of statistical significance regarding RTD comparisons might be due to a lack of statistical power. Therefore, we establish the small sample size as the fifth limitation in our study.

## Abbreviations

Ca^2^^+^: Calcium
ETorque: Explosive torque
EMG: Electromyography
H^+^: Hydrogen ions
H_2_PO_4-_: Dihydrogen phosphate
MVIC: Maximum voluntary isometric contraction
PA: Physical activity
Pi: Inorganic phosphate
pRTD: Peak rate of torque development
PT: Peak torque
RTD: Rate of torque development
